# SSTDhunter: a curated gene database for investigating androgen producing potential in microbiota species

**DOI:** 10.3389/fcimb.2026.1754671

**Published:** 2026-01-23

**Authors:** Shaojing Wang, Yifan Yang, Li Lei, Rongxin Wan, Zhaoying Su, Yan Liu, Huiqin Tang, Guoying Hu, Changlin Li, Changying Li, Jinhuan Meng, Kuo Yang

**Affiliations:** 1 Tianjin Institute of Urology, The Second Hospital of Tianjin Medical University, Tianjin, China; 2 Cheeloo College of Medicine, Shandong University, Jinan, Shandong, China; 3 Collection Management Department, Natural History Museum of China, Beijin, China

**Keywords:** androgens, gut microbiome, prostate cancer, SSTDhunter database, steroid-17,20-desmolase

## Abstract

Androgens are critical for the growth of prostate cells, as well as prostate tumor cells. For prostate cancer patients under Androgen Deprivation Therapy (ADT) such as castration treatment, investigating the potential for androgen production by gut microbes is crucial. In microbe species, the side chain cleavage activity of steroid-17, 20-desmolase (SSTD) is responsible for 11-oxy-androgens production by biotransformation from cortisol, as well as from other endogenous steroids and pharmaceutical glucocorticoids. The side-chain cleavage product of prednisone could significantly promote the proliferation of prostate cancer cells. The SSTD is a complex formed by N-terminal and C-terminal transketolases encoded by *desA* and *desB* genes, whose activity has been well-characterized in *Clostridium scindens* ATCC 35704. While a void still existed in evaluating the androgen producing potential by gut microbiota owing to relatively low abundance of SSTD-carrying species and the lack of professional gene database. Meanwhile, mining SSTD encoding genes in explosion sequencing data has become computationally expensive and time-consuming using comprehensive database. Here, a professional database consisted of SSTD-coding genes, named SSTDhunter, was constructed using a large-scale genomic analysis along with homologous genes as background. These SSTD-coding genes were reconstruction through comprehensive characteristics consisted of operon structures, sequence identities, phylogenetic topologies and comparative analysis. To reduce false positives, protein sequences of homologous genes *tktA*, which encode component of sugar transketolase, were also included in SSTDhunter database as background noise. SSTDhunter is for rapid investigation of SSTD-coding genes in massive metagenomic data, which is freely available at http://www.orgene.net/SSTDhunter/.

## Introduction

Transketolase (EC 2.2.1.1) catalyzes the transfer of a two-carbon keto group from ketose sugar to aldose sugar, which is critical and conserved across life forms (Josephson and Fraenkel, 1969; Josephson and Fraenkel, 1974). In *Escherichia coli*, two transketolase isoforms with 75% amino acid identity were identified, namely *tktA* (transketolase 1) and *tktB* (transketolase 2) (Iida et al., 1993; Zhao and Winkler, 1994). The TktA is responsible for the major transketolase activity while the *tktB* gene encodes a minor transketolase activity (Iida et al., 1993; Zhao and Winkler, 1994). The two genes located distantly on chromosome (Blattner et al., 1997), i.e. *tktA* gene located on reverse strand from 3, 079, 644bp to 3, 081, 635bp, while *tktB* gene located on forward strand from 2, 579, 636bp to 2, 581, 639bp in genome of *Escherichia coli* str. K-12 (ASM584v2).

The SSTD activity, which is responsible for the side-chain cleavage of glucocorticoids (i.e., 11-oxy-androgens production by bacteria), was firstly described in *C. scindens* ATCC 35704 in the 1980s as a special subtype of transketolas (Winter et al., 1984; Morris et al., 1985; Krafft et al., 1987). This strain was isolated from human gut microbiota based on its ability of cortisol side-chain cleavage. The *C. scindens* isolates can be cultured under strict anaerobic conditions at 37 °C and pH 6.5–7 in enriched media. As part of the Human Microbiome Project (HMP), the genome of *C. scindens* ATCC 35704 was determined in 2010 (GenBank ID: ASM15450v1, comprise 3.7 Mb genome size with G+C content of 46%), which provided genomic knowledgebase for this SSTD carrying species. The SSTD is a complex formed by DesA and DesB subunits, which are annotated as N-terminal and C-terminal transketolases, respectively (Devendran et al., 2017; Devendran et al., 2018). Different from *tktAB*, the encoding genes of SSTD (i.e., *desA* and *desB*) located adjacently in operon. The SSTD operon was inducible by cortisol (i.e., upregulated ~1, 000-fold), which was identified using a RNA-seq approach (Ridlon et al., 2013). Beside *desAB*, the SSTD operon in *C. scindens* ATCC 35704 includes *desC* and *desD* genes, which encode 20ɑ-HSDH and a putative cortisol transporter, respectively (Ridlon et al., 2013; Devendran et al., 2018). Subsequent investigations of SSTD-coding genes across a dozen strains suggested the rarely existence of SSTD activity in *C. scindens* species (Ridlon et al., 2013; Ly et al., 2020; Wang et al., 2024), which was confirmed by a most recent pan-genome analysis of *C. scindens (*Olivos-Caicedo et al., 2024). Beside SSTD operon consisted of four genes (i.e., *desABCE*) in *C. scindens*, another type of SSTD operon consisted of three genes (i.e., *desEAB*) was identified in *Butyricicoccus desmolans*, *Clostridium cadaveris* and *Propionibacterium lymphophilum*, which include *desE* gene encoding for 20β-HSDH. Although these *desEAB* operons were recovered using sequence similarity analysis strategy, they were also considered responsible for 11-oxy-androgens production (Devendran et al., 2017; Ly et al., 2020).

Prostate cancer is one of the most common malignant tumors in male population. The proliferation of prostate tumor cells is typically driven by androgen-dependent mechanisms. Since the 1940s, ADT has been the cornerstone of prostate cancer treatment, with well responses in early stage but later progressing into castration-resistant prostate cancer (CRPC) (Choi et al., 2022). However, androgens produced by gut microbes can undermine the clinical benefit of ADT (Ly et al., 2021). A recent study confirmed that 11-oxy-androgens produced by *C. scindens* ATCC 35704 resulted in the proliferation of LNCaP cells (Bui et al., 2023). Beside endogenous steroids, glucocorticoid drugs could also be side-chain cleaved by SSTD activity (Zimmermann et al., 2019; Ly et al., 2020). The recombinant enzyme DesAB exhibited high catalytic activity against prednisone, and the metabolism product also significantly promoted LNCaP cells’ proliferation (Ly et al., 2020). Fecal microbiota transplantation (FMT) further confirmed the contribution of gut microbiome to prostate cancer progression (Pernigoni et al., 2021; Hsiao et al., 2023). When feces from CRPC patients and healthy adults were transplanted into prostate cancer mouse models undergoing ADT, mice receiving CRPC feces developed CRPC more rapidly (Pernigoni et al., 2021).

The later CRPC develops, the greater benefit patient can derive from ADT. Evaluating the androgen producing potential by gut microbiota can provide a basis for clinical intervention in prostate cancer progression. However, the *C. scindens* species are reported of relatively low abundance in gut microbiome (Berr et al., 1996). Meanwhile SSTD activity is rarely reported within limited taxonomic lineages (Ly et al., 2020). Limited knowledgebase resulted in lack of effective way for evaluating the androgen producing potential of gut microbiota. In this study, we applied the database investigation strategy to fill this void. A professional gene database of steroid-17, 20-desmolase was constructed using a large-scale genomic analysis along with homologous genes as background. The database was designed as SSTDhunter, with further investigation of SSTD-carrying species (i.e., niches distribution, taxonomic lineage and Horizontal Gene Transfer (HGT)) and its application in rapid investigation of SSTD-coding genes in several metagenomic datasets.

## Materials and methods

### Identification of SSTD operons

The protein sequences of the coding genes in *desABCD* operon of *C. scindens* (i.e., WP_004606448.1, WP_004606449.1, WP_004606450.1 and WP_004606451.1) and in *desEAB* operon of *C. cadaveris* (i.e., WP_027640050.1, WP_027640052.1 and WP_027640053.1) were collected for initial database construction. All available prokaryotes genomes (both bacteria and archaea) were retrieved for SSTD operons identification. The genomes representing for at least 220 phyla (**Supplementary Table S1**) was downloaded from NCBI (www.ncbi.nlm.nih.gov/datasets/genome/) on Jul 20, 2024. More detailed, the dataset included over 2.23 million genome sequences from isolates or metagenome-assembled genomes (MAGs), representing for 84, 044 species across 4, 918 genera. Taxonomically, archaea genomes accounted for 1.08% (24, 154/2, 238, 298), while bacteria genomes made up to 98.92% (2, 214, 144/2, 238, 298) of the dataset. Gene prediction was performed using Prokka v1.13 (Seemann, 2014). Candidate SSTD operons were identified by screening for the presence of *desAB* genes using blastp in BLAST package v2.9.0 (Camacho et al., 2009a) with an e-value threshold of 1e-10. The presence of adjacent *desAB* genes was the initial criterion for SSTD operons identification.

DNA segments flanking the *desAB* genes, ranging from 5 kb upstream to 10 kb downstream of *desA* start codon, were extracted from genome sequences for further operon organization validation. These fragments were re-annotated using the Prokaryotic Genome Annotation Pipeline (PGAP) v.2022-10-03.build6384 (Tatusova et al., 2016). The predicted genes were further screened for *desABCDE* identification through blastx in BLAST package v2.9.0 (Camacho et al., 2009a) with an e-value threshold of 1e-10. The organization of SSTD operons was generated using R v4.1.2 (www.r-project.org) with ggplot2 and gggenes packages.

The Maximum Likelihood (ML) phylogenetic trees were reconstructed separately for *desA* and *desB* from SSTD operons. The gene sequences were aligned by MAFFT v7.407 (Katoh and Standley, 2013). Subsequently, the ML trees were constructed using the RAxML-NG v.1.1 (Kozlov et al., 2019), with 1000 rapid bootstrap. The best fitting model of “TIM2+I+G4” was determined by ModelTest-NG v0.1.7 (Darriba et al., 2020). The final tree was midpoint-rooted and visualized using iTOL (Letunic and Bork, 2021). The Sankey diagram was generated using R v4.1.2 (www.r-project.org) with ggplot2 and ggalluvial packages.

### Database construction

The “SSTDhunter” was designed for accurate and rapid investigation of SSTD profiles in metagenomic data, which consists of protein coding genes sequences and associated tool. All encoding sequences of DesAB in operon were retained in SSTDhunter. To reduce false positives, homologous genes sequences were also added into the database. Considering the similarity of *desAB* for SSTD and *tktAB* for transketolases, *tktA* gene sequences were merged into SSTDhunter for false positive elimination, using a best-hit strategy based on BLAST. The 50 most diverse *tktA* gene sequences were obtained in Conserved Domain Database (CDD) from NCBI (www.ncbi.nlm.nih.gov) under COG0021. The associated tool, named SSTDhunter.pl, was written in Perl for generating the abundance of SSTD-encoding genes in RPKM.

### Diversity analysis of SSTD-carrying microbes

The ecological niches and geographic data for SSTD-carrying microbes were retrieved from the NCBI website according to their corresponding assembly accession IDs. The taxonomic lineages were reassigned using GTDB-Tk v.2.1.1 (Chaumeil et al., 2022), based on the Genome Taxonomy Database (GTDB) release R207_v2 (Parks et al., 2022). The ML species tree was constructed using concatenated sequences of 120 bacterial marker genes generated by GTDB-Tk, using RAxML-NG v.1.1 (Kozlov et al., 2019)with 1000 bootstrap replicates. The best fitting model of “LG+I+G4+F” was determined by ModelTest-NG v0.1.7 (Darriba et al., 2020). The final tree was midpoint-rooted and visualized with iTOL (Letunic and Bork, 2021). Genome-wide average amino acid identity (AAI) was calculated using EzAAI v1.1 (Kim et al., 2021). Distribution of the geographic location and the AAI values of SSTD-carrying microbes were visualized in R v4.1.2 (www.r-project.org) using the ggplot2 package.

### Metagenomic analysis

Gut metagenomic data of 314 samples were downloaded from the ENA database of European Bioinformatics Institute (www.ebi.ac.uk/ena/browser/home) under study accession numbers of PRJNA749645 (74 samples), PRJDB18316 (166 samples) and PRJNA1076083 (74 samples). The unassembled raw datasets were downloaded in FASTQ format. These raw reads were filtered by Trimmomatic (Bolger et al., 2014) v0.32 for a minimum quality of 20 and a minimum read length of 50bp. Contamination reads from host were removed using HoCoRT (Rumbavicius et al., 2023). The “SSTDhunter” was applied for the investigation of *desABCDE* genes. Briefly, the reads were aligned to SSTDhunterDB using Bowtie2 (Langmead and Salzberg, 2012) with default parameters. Subsequently, the output files in SAM format were processed by SSTDhunter.pl to generate the abundance of each gene in RPKM. The difference in *desAB* gene abundance in the gut microbiota between prostate cancer patients and non-cancer individuals was analyzed using Wilcoxon rank-sum test, with a significance threshold of *P* < 0.05. The effect sizes were also estimated using rank-biserial correlation approach.

Samples in PRJNA749645, which was previously reported for revealing the contribution of gut microbiota to endocrine resistance in CRPC (Pernigoni et al., 2021), were assembled for further analysis. The metaWRAP pipeline was applied for generating contigs and MAGs (Uritskiy et al., 2018). Briefly, the modules of “assembly”, “Binning” and “Bin_refinement” were performed step by step with default parameters. Those MAGs with completeness ≥50% and contamination <10% were retained for downstream analyses. Gene calling of these assembled contigs and MAGs were applied by Prodigal (Hyatt et al., 2010) v2.6.3 with the parameter “-p meta”. The amino acid sequences of predicted genes were searched against SSTDhunterDB using the tblastn tool of BLAST (Camacho et al., 2009b) package with parameters “-evalue 1e-10 -outfmt 6”. The best hits with local identity > 50% were retained. Only samples with the existence of both *desA* and *desB* genes were retained for comparison.

## Results

### SSTD operons investigation and genetic diversity analysis

**Figure 1:**
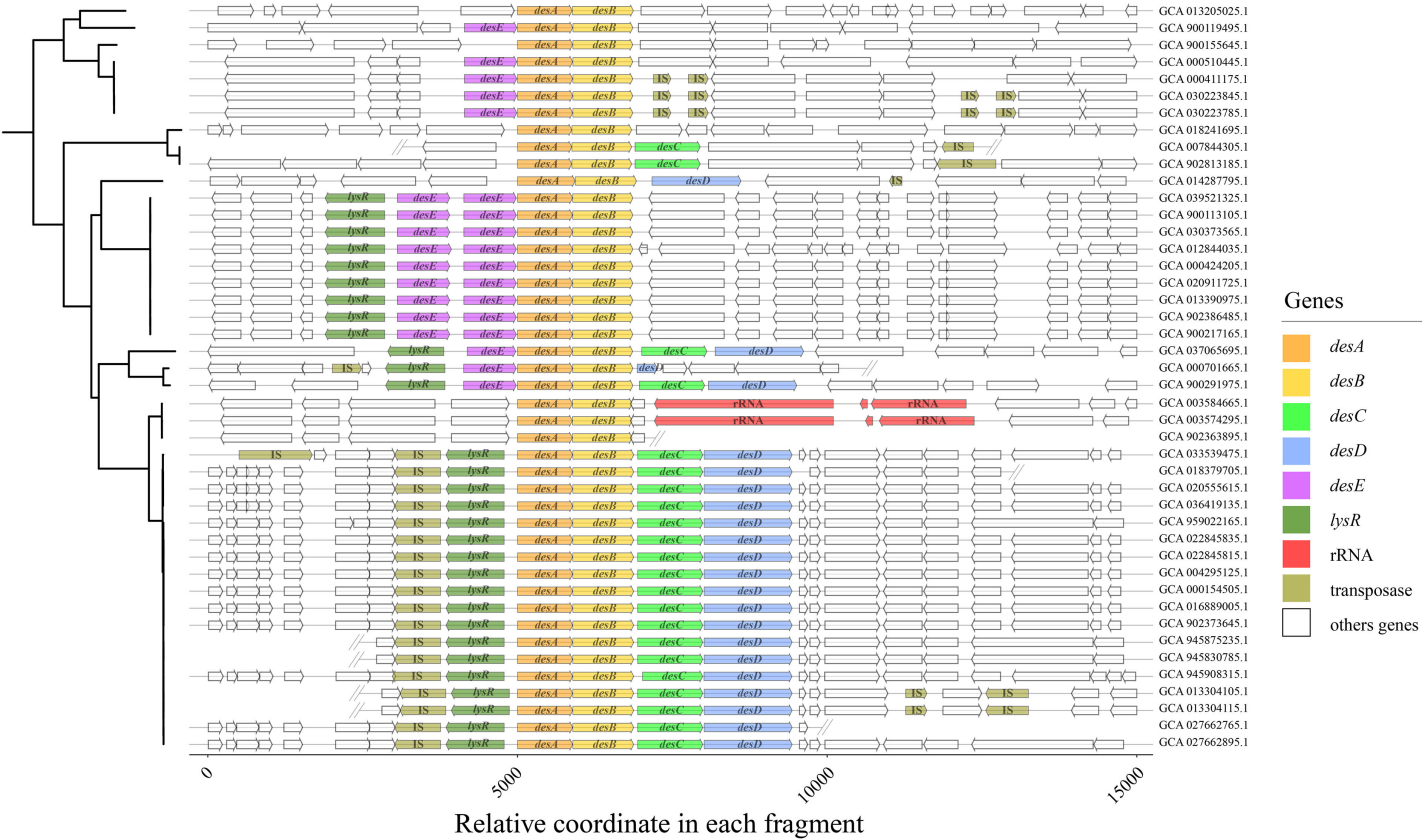
The organization of SSTD operons. DNA fragments were aligned according to the starting code of *desA*. The double slash represented for truncated contigs.

Over 2.23 million prokaryotic genomes were retrieved from NCBI for the investigation of SSTD operons. A total of 44 SSTD operons were observed (**Figure 1**, **Supplementary Table S2**). And the nucleotide sequences of *desAB* and its flanking regions were extracted from the corresponding genomes to address the diversity of SSTD operons. Both operon types of *desABCD* and *desEAB* were observed. As inferred from **Figure 1**, 15 *desEAB* operons, 18 *desABCD* operons and 2 mixed operons of *desEABCD* were observed, with the remaining 9 operons truncated or incomplete. Genes encoding for transposases and LysR-type transcriptional regulators were located adjacent to these operons, suggesting a coordinated regulatory mechanism in which the LysR regulator controls operons expression, potentially in response to the presence or activity of the associated mobile genetic element (the transposon).

**Figure 2:**
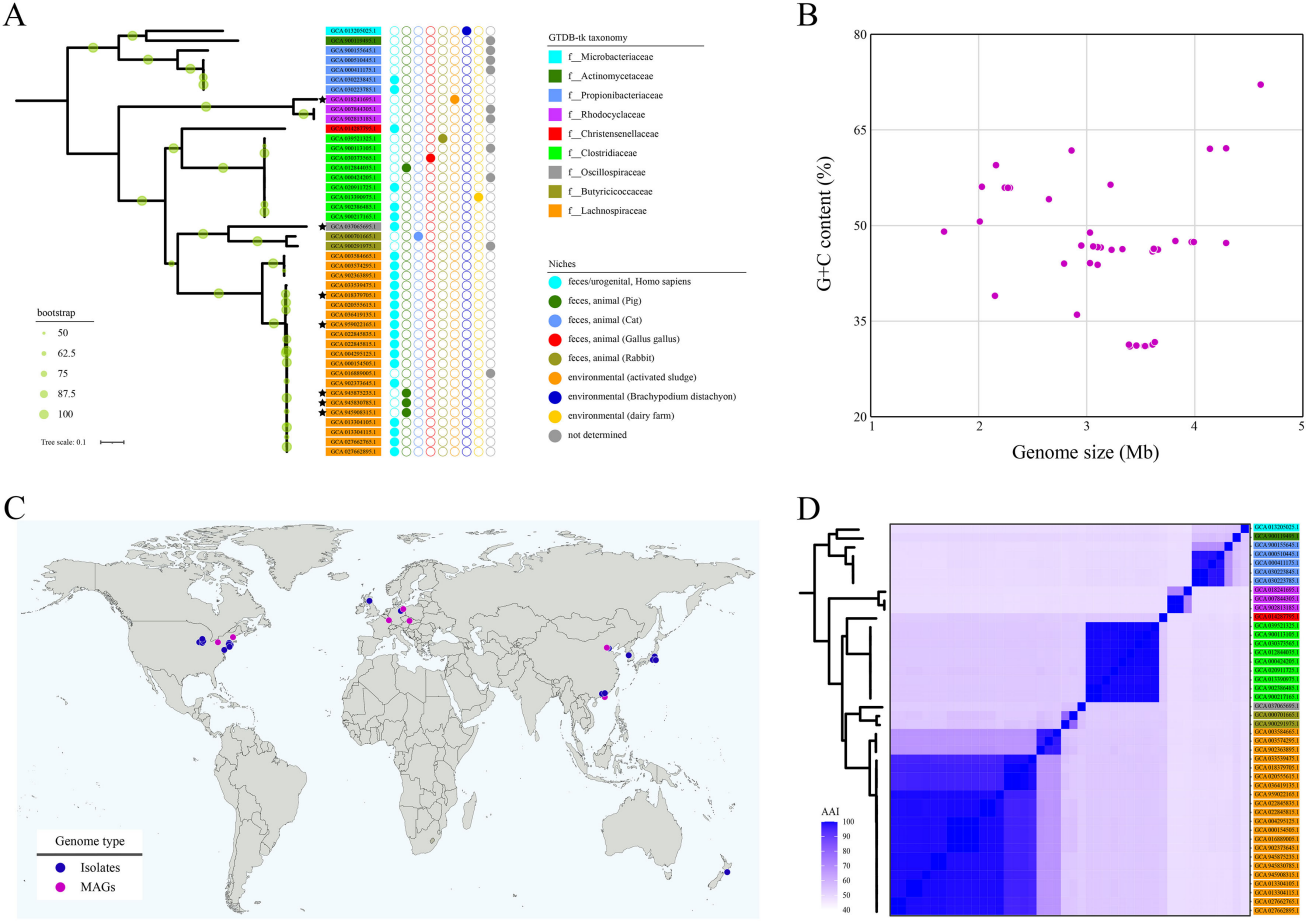
Distant relationships among SSTD-carrying microbes **(A)** Phylogenetic tree of SSTD-carrying bacteria. The ML tree was midpoint rooted. The leaf labels represent the accession numbers of each genome within NCBI genome database, with stars indicating MAGs. The background colors of the leaf labels reflect the taxonomy as inferred by GTDB-tk. The bubble diagram on the right illustrates the niches from which these SSTD-carrying microbes were sourced. **(B)** Genome size and G+C content distribution of SSTD-carrying microbes. **(C)** The geographic distribution of SSTD-carrying microbes inferred from sample collection sites. **(D)** Genome-wide AAI distribution of SSTD-carrying microbes.

All of the 44 operons are existed as single copy in each corresponding bacterial genomes, of which 84.09% (37/44) were sourced from cultured strains and 15.91% (7/44) were from MAGs (**Supplementary Table S3**). These bacterial species distributed across nine families within three major phyla (i.e., Firmicutes, Actinobacteriota, and Proteobacteria), representing for at least 14 species (**Figure 2A**). No SSTD operon was observed in any archaea genome; even so, the taxonomic lineage distribution suggested a potential high diversity in SSTD-carrying microbes. The diversity of SSTD operons organization was correlated with taxonomic lineage, where species with closer genetic relationships exhibited similar operon structures. According to the sampling environments of these genomes, SSTD-carrying microbes were inferred to have a broad niches adaptability, representing for eight different niches (**Figure 2A**). These niches were categorized into three types: human hosts, other animal hosts, and environmental sources. Besides Homo species, the SSTD-carrying microbes were observed within feces of four different animal species. Interesting, SSTD-carrying microbes were also observed in environmental associated with activated sludge, dairy farm and plant (i.e., *Brachypodium distachyon*). No bias towards niche adaptation reflected in the phylogenetic tree was observed. The genome features of these SSTD-carrying microbes varied greatly, with genome sizes ranging from 1.68Mb to 4.61Mb and G+C content ranging from 31.06% to 72.11% (**Figure 2B**). According to the sample collection sites, SSTD-carrying microbes are distributed almost worldwide, including in Asia, Europe, the Americas, and other regions (**Figure 2C**). The genome-wide AAI values also revealed distant relationships among these SSTD-carrying microbes, with an average AAI value of 61.88% ± 18.25, and the lowest AAI value of 47.91% observed between strains (**Figure 2D**).

**Figure 3:**
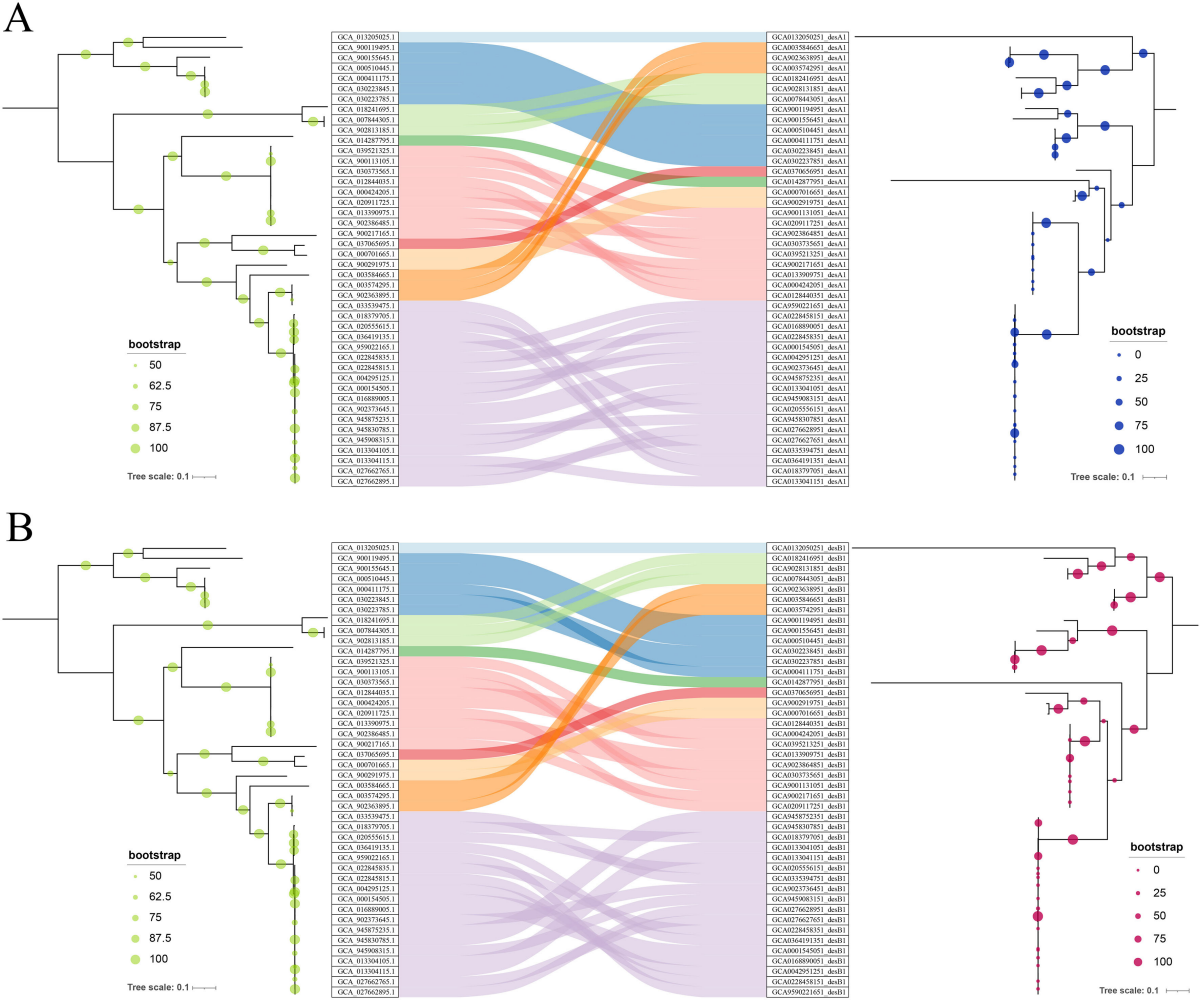
Comparative phylogenetic analysis **(A)** Conflict phylogenetic relationship between *desA* genes and their corresponding genomes. The tree on the left represents the phylogenetic relationship of species, while the tree on the right depicts the phylogenetic relationship of *desA* genes. The Sankey diagram in the middle revealed HGTs of *desA* gene between distant species. **(B)** Conflict phylogenetic relationship between *desB* genes (right) and their corresponding genomes (left) revealed HGTs of *desB* gene between distant species.

As mentioned above in **Figure 1**, the dissemination of SSTD operon is considering associated with transposases, which might responsible for HGTs of these operons between distant species. A comparative phylogenetic analysis was applied for further analysis. The ML trees of both *desA* and *desB* genes were reconstructed. Conflict phylogenetic relationship between *desA* genes and their corresponding genomes revealed HGTs of SSTD operons between distant species (**Figure 3A**). E.g., *Mediterraneibacter butyricigenes* (NCBI accession No. of GCA_003584665.1) exhibits a close phylogenetic relationship to *C. scindens* (i.e., GCA_022845835.1) within the family Lachnospiraceae, while its *desA* gene is more closely related to that of *Denitratisoma oestradiolicum* (i.e., GCA_007844305.1), a distant species from the family Rhodocyclaceae. The consistent conclusion was also inferred from the comparative phylogenetic analysis of *desB* (**Figure 3B**).

### Construction of gene database for SSTD investigation

**Figure 4:**
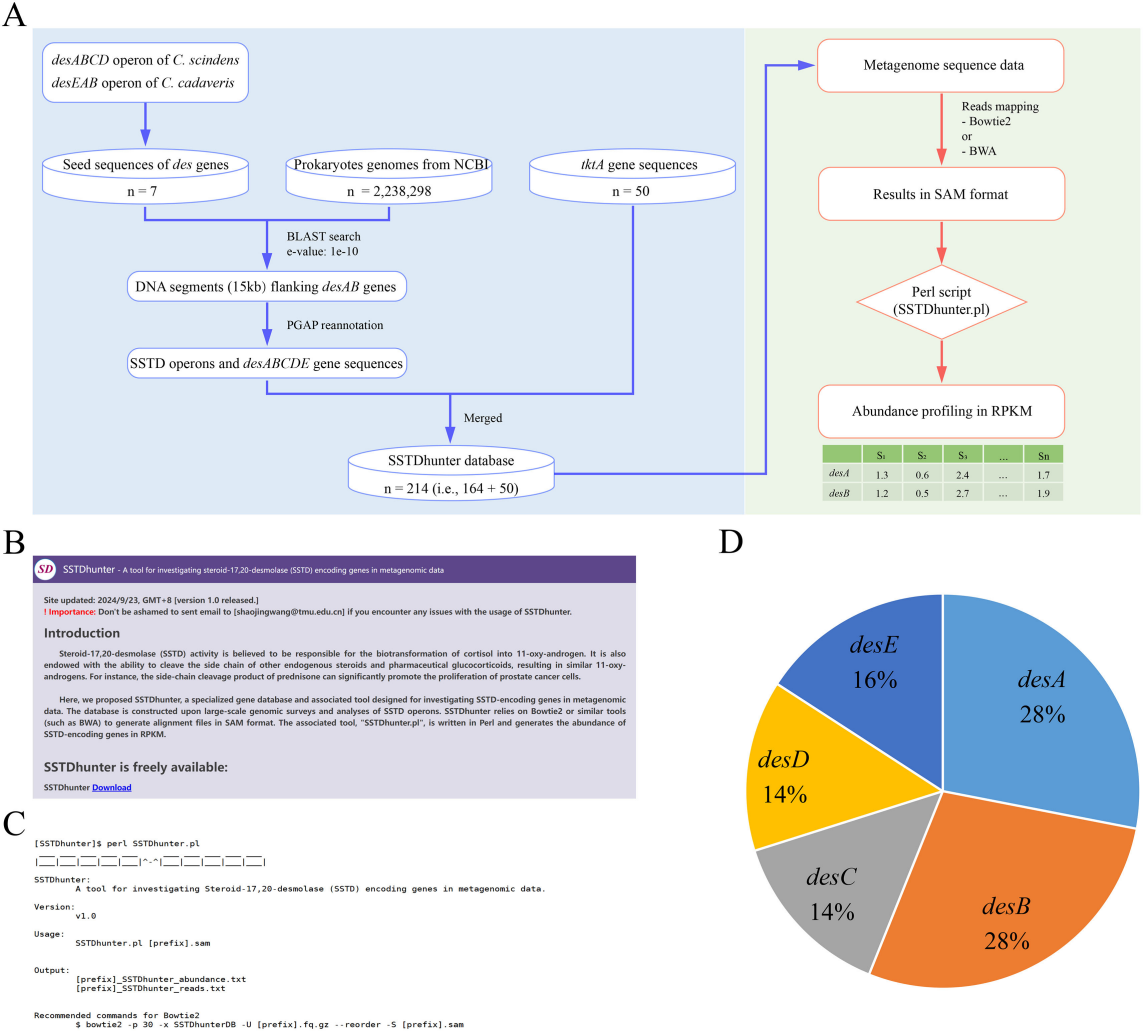
Database description (**A**) Schematic diagram of the SSTDhunter workflow **(B)** Web interface of SSTDhunter. **(C)** Usage description of SSTDhunter.pl. **(D)** Composition of *desEABCD* in SSTDhunterDB.

A professional gene database for fast investigation of SSTD in massive datasets, named SSTDhunter, was designed based on the results of SSTD operons above (**Figure 4A**). SSTDhunter is a package consisted of one gene database and one Perl script. The gene database, named SSTDhunterDB, consisted of 164 nucleotide sequences of *desABCDE* retrieved from SSTD operons (**Figure 4B**). The associated script, named SSTDhunter.pl, was written in Perl for generating the abundance of SSTD-encoding genes in RPKM, along with the corresponding reads in FASTA format (**Figure 4C**). SSTDhunter is freely available at http://www.orgene.net/SSTDhunter/. Briefly, the *desA* and *desB* genes are most abundant with proportion of 28% for each. The proportion of *desC*, *desD* and *desE* were 14%, 14% and 16%, respectively (**Figure 4D**).

**Figure 5:**
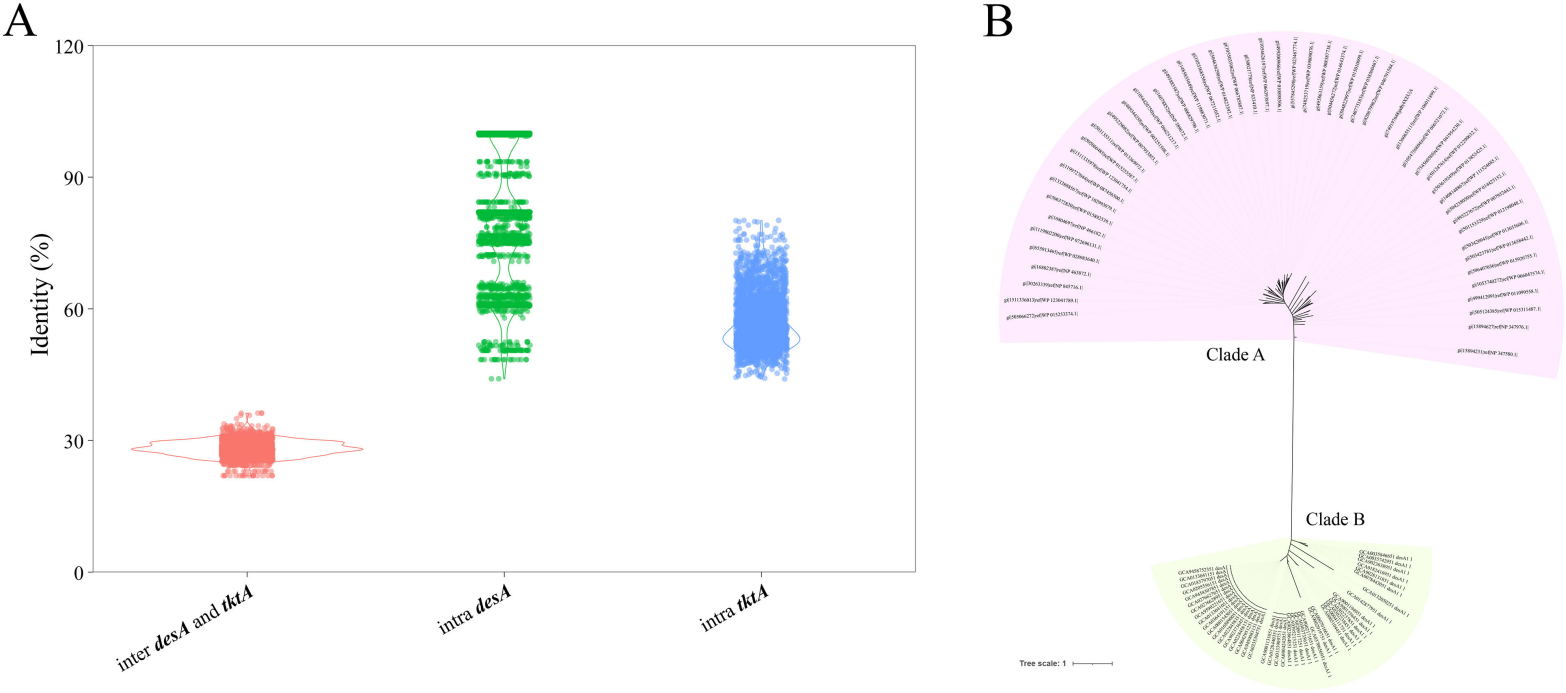
Comparation of *desA* and *tktA* genes **(A)** The pairwise identities of *desA* and *tktA* sequences **(B)** The phylogenetic analysis of *desA* and *tktA* genes.

The encoding genes of SSTD (i.e., *desAB*) is somewhat of similarity to sugar transketolases, which might cause false positives. The consideration of the genes *tktA* and *tktB*, which encode sugar transketolases, is essential for the investigation of *desAB*. The pairwise identities of *desA* and *tktA* sequences were calculated. Notably, lower pairwise identities were observed between *desA* and *tktA* genes, compared to the pairwise identities of intra- *desA* and *tktA* genes (**Figure 5A**). The average pairwise identity between *desA* and *tktA* genes was 28.19% ± 1.90%, while the average pairwise identities of intra- *desA* and *tktA* genes were 78.08% ± 14.78% and 57.69% ± 6.45%. The phylogenetic analysis of *desA* and *tktA* genes revealed two distant mono-clades, representing for the two genes, respectively (**Figure 5B**). The results suggested that these two types of genes can be distinguished based on their sequences. And the protein sequences of *tktA* were also retained in SSTDhunterDB as background noise for false positives reduction.

### Investigation of SSTD-coding genes in metagenomes

**Figure 6:**
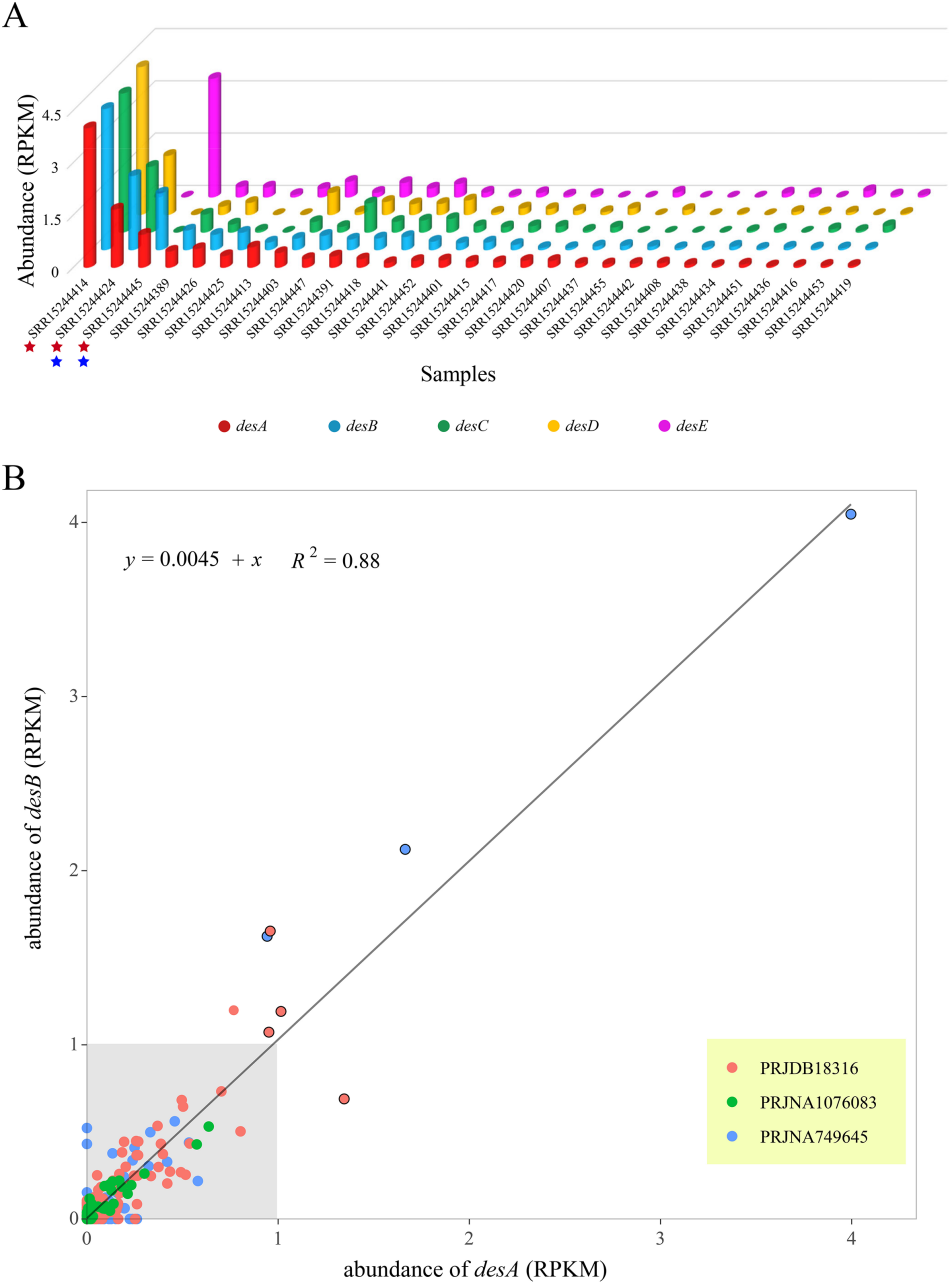
Investigation of SSTD-coding genes in Metagenomes **(A)** Comparation of SSTDhunter pipeline and classic assembly & binning pipeline. Only samples containing both *desA* and *desB* genes were presented. The red stars at the bottom indicated the existence of *desAB* genes in the contigs of corresponding samples, while blue stars indicated the presence of *desAB* genes in the MAGs from corresponding samples. **(B)** Consistent abundance of *desA* and *desB* genes. The majority of the *desAB* genes with RPKM lower than 1 was marked using gray background. Beyond the gray background, dots marked with black cycles represented for samples originated from prostate cancer patients.

To validate the performance of SSTDhunter, a previously reported metagenomic dataset under NCBI accession numbers of PRJNA749645 was applied for SSTD investigation using both of the SSTDhunter pipeline and the classic assembly & binning pipeline (**Supplementary Table S4**). In total, 74 gut microbiota samples of prostate cancer patients were included in this dataset. Using the SSTDhunter pipeline, 39.19% of these samples (i.e., 29 out of 74) were observed to harbor both *desA* and *desB* genes, forming either candidate *desABCD* or *desEAB* operons (**Figure 6A**). While using the classic pipeline, the *desAB* genes were detected only in contigs of three samples with the highest abundance and in MAGs of the second and third most abundant samples (**Figure 6A**). The *desAB* genes were missed detection in contigs and MAGs of these samples with low *desAB* abundance. These results clearly indicated that SSTDhunter pipeline offers exceptionally high resolution. Moreover, SSTD investigation using predicted genes from assembled contigs and MAGs is a more complex process with high computational costs. On the contrary, the SSTDhunter pipeline is independence from genome assembly, binning and gene prediction, which can significantly accelerate process of large-scale data analysis.

To gain a deeper understanding of SSTD distribution, the SSTDhunter pipeline was applied to a much larger dataset, encompassing raw metagenomic data of 3.29TB from 314 individuals (**Supplementary Table S4**). This metagenomic dataset was obtained from European Bioinformatics Institute under accession numbers of PRJNA749645 (74 individuals), PRJDB18316 (166 individuals) and PRJNA1076083 (74 individuals), representing for cohorts from Switzerland, Japan, and China, respectively. The result suggested that SSTD existed in 41.08% (129 out of 314) of these samples. The abundance of *desA* and *desB* genes exhibited high consistency (**Figure 6B**). Although widely distributed, SSTD typically existed at very low abundances. The majority of these SSTD positive samples (95.62%, 175 out of 183) exhibited RPKM of *desAB* genes both lower than 1. The *desAB* genes with RPKM higher than 1 were only observed in 8 samples, 7 out of which were originated from prostate cancer patients (**Figure 6B**). Thus, the *desAB* genes exhibited characteristics of widespread but relatively low-abundance was concluded, which may be one reason they had long been neglected. Furthermore, the average abundance of *desA* and *desB* genes in gut microbiota of prostate cancer patients were 0.152 and 0.159, respectively. In contrast, the average abundance of *desA* and *desB* genes in gut microbiota of non-cancer individuals were 0.063 and 0.071, respectively. For *desA* gene, the abundance was significantly higher in the gut microbiota of prostate cancer patients than in non-cancer individuals (Wilcoxon rank-sum test, *p* = 0.0072), with a small effect size (rank-biserial correlation = 0.15) and a modest shift in distribution (median difference = 1.0 × 10^-6^, 95% CI: 4.9 × 10^-5^ - 1.9 × 10^-^²). The abundance of *desB* gene also significantly higher in the gut microbiota of prostate cancer patients than in non-cancer individuals (Wilcoxon rank-sum test, *p* = 0.041), with a small effect size (rank-biserial correlation = 0.12) and a subtle shift in distribution (median difference = 3.6 × 10^-5^, 95% CI: −1.2 × 10^-5^ - 1.2 × 10^-^³).

## Discussion

In our present study, the widest possible diversity of SSTD was investigated across over 2.23 million genomes. And a novel higher diversity was revealed from multiple perspectives, including geographic distribution, niches adaption, species diversity, and genetic diversity. Based on the results of SSTD operon and phylogenetic analyses, SSTDhunter was designed for the accurate and rapid investigation of steroid-17, 20-desmolase profiles in massive metagenomic data.

The SSTD had previously been reported for its taxonomically rare and niche-specific within the human gut and urogenital microbiomes, supported by large-scale phylogenetic analysis of DesA (Ly et al., 2020). The number of observed SSTD-carrying genomes in this study was relatively small, consistent with previous view of its taxonomic limitations, even rare occurrences within species of *C. scindens (*Ridlon et al., 2013; Wang et al., 2024). Besides this agreement, speculation regarding potential higher diversity had been raised despite the limited number of observed genomes. Geographic distribution of the corresponding samples for these genomes revealed globally dissemination of SSTD-carrying microbes. Though limited within three major phyla, these SSTD-carrying microbes distributed in at least 15 species from nine families. Together with the distant relationships revealed by the phylogenetic tree, insufficient investigation of SSTD pathway was indicated, suggesting that there may be unobserved SSTD-carrying taxonomic lineages. Furthermore, several clear HGT events was observed through phylogenetic conflicts, providing novel insights into the potential dissemination of the SSTD pathway across distant species. Horizontal transfer of large DNA fragments, e.g. operons, depends on mobile elements. Positive correlation between the abundance of mobile genetic elements and the frequency of HGT were generally observed (Springael and Top, 2004). Although the observation of numerous IS elements in flanking regions suggested their potential role in the mobilization, the mechanisms underlying the horizontal transfer of SSTD operons between species remained a mystery and further researches were required.

Technically, metagenomics offers the advantage of conducting microbial investigation independent from isolation and culture. However, only 19.57% (9 out of 46) of these SSTD-carrying microbes were inferred from MAGs, mainly located in the family Lachnospiraceae. The results suggested a limited effectiveness of MAGs in detecting low-abundance microbes, such as *C. scindens* and other SSTD-carrying species, likely due to the challenges of assembly and binning from relatively low reads abundance in raw datasets. To verify this hypothesis, gut metagenomic datasets from feces samples of 74 prostate cancer patients were further analyzed, investigating SSTD-coding genes at three levels: raw reads, assembled contigs, and binning-derived MAGs. The results showed that mapping methods revealed the existence of SSTD-coding genes in the raw reads of 29 samples, although most samples harbored relatively low abundances of these genes. In contrast, SSTD-coding genes were detected in only three samples based on contigs and MAGs, which exhibited the highest abundances of these genes among all samples. These findings confirmed the feasibility and effectiveness of mapping methods for SSTD-coding genes investigation.

With the rapid advancement of sequencing technologies, metagenomic sequencing data have increased exponentially, placing extremely demanding requirements on computational resources. Although several pipelines based on well-annotated comprehensive gene catalogs are currently available, investigations the *desAB* genes remain excessively time-consuming and, in some cases, cannot be completed within a reasonable timeframe. Therefore, the SSTDhunter tool was designed and released, aimed at providing a rapid, convenient, and effective way for investigating SSTD-coding genes directly from large metagenomic raw reads datasets. RPKM normalizes read counts by gene length and sequencing depth and is commonly used to estimate relative gene abundance in transcriptomics studies. Considering that both *desA* and *desB* are present as single-copy genes in the genome, RPKM was applied to assess the relative abundance of *desAB*-carrying microorganisms in metagenomic datasets. Future studies should also consider potential copy number variation of the *desAB* genes, as this may necessitate further refinement of the current strategy. Although SSTDhunter exhibit advanced characteristics (i.e., user-friendliness, efficiency, and clarity of results), it also had limitations, such as the inability to infer the microbial hosts of these genes. However, hosts information can be provided according to taxonomic lineages inferred from MAGs. Fortunately, the epicPCR (Emulsion, Paired Isolation and Concatenation PCR) (Roman et al., 2021) was discovered. EpicPCR is a recent single-cell genomic method based on a fusion-PCR that allows us to link a functional sequence of interest to a 16S rRNA gene fragment. This method enables mass sequencing of the resulting amplicons for taxonomic assignment of the functional sequence-carrying bacteria. Based on the gene sequences provided by SSTDhunter, a specialized epicPCR system could be designed for investigating the microbial hosts of SSTD-coding genes. While epicPCR exhibited advantages for investigating the dissemination of functional genes across species, their abundance information was missed. Therefore, a more detailed and accurate characterization of SSTD-coding genes can be achieved through the combined application of SSTDhunter and epicPCR in future. This integration will offer a deeper understanding of SSTD-carrying microbes, particularly those species that have not been isolated and cultured yet.

Based on metagenomic analyses, increased abundance of microorganisms harboring the *desAB* genes suggested an overall enhancement of the androgen-producing potential of the microbial community. Therefore, the variations in *desAB* abundance at the DNA level likely reflect changes in the abundance of microbial taxa carrying this gene. This observation suggests the potential for functional activity; however, it does not directly indicate higher gene expression levels or increased production of downstream metabolites. According to these results represented in this study, the overlooked importance of bacterial-derived androgens was highlighted through the investigation of wider spread distribution of SSTD-coding genes. Therefore, gut microbiome intervention might be a potential strategy to delay the progression of Hormone-Sensitive Prostate Cancer (HSPC) to CRPC. These publicly available metagenomic datasets applied in this study were derived from three independent cohorts, and therefore differ in geographic origin, disease status, and treatment background. Although a higher abundance of the *desAB* genes was observed in samples from prostate cancer patients, the results were drawn from a pooled comparison across cohorts without considering the geographic or cohort-specific variations. More comprehensive datasets with richer metadata will be required in future studies to validate these findings. Future studies were still required to validate this conclusion using systematically designed datasets. Furthermore, in-depth exploration of androgen synthesis-related functional genes in the gut microbiome could provide a theoretical basis for understanding the interaction between gut microbiome and prostate cancer patients, thereby extending the effectiveness of ADT and ultimately improving patients’ quality of life.

## Data Availability

The data presented in the study are deposited in the GitHub repository at https://github.com/shaojingwang2018/SSTDhunter, the version number is v1.0.0.
